# Frog foams and natural protein surfactants

**DOI:** 10.1016/j.colsurfa.2017.01.049

**Published:** 2017-12-05

**Authors:** Alan Cooper, Steven J. Vance, Brian O. Smith, Malcolm W. Kennedy

**Affiliations:** a School of Chemistry, University of Glasgow, Glasgow, G12 8QQ, UK; b Institute of Molecular, Cell & Systems Biology, College of Medical, Veterinary and Life Sciences, University of Glasgow, G12 8QQ, UK; c Institute of Biodiversity, Animal Health and Comparative Medicine, College of Medical, Veterinary and Life Sciences, University of Glasgow, G12 8QQ, UK

**Keywords:** Surface tension, Ranaspumin, Ranasmurfin, Latherin, Protein structure

## Abstract

Foams and surfactants are relatively rare in biology because of their potential to harm cell membranes and other delicate tissues. However, in recent work we have identified and characterized a number of natural surfactant proteins found in the foam nests of tropical frogs and other unusual sources. These proteins, and their associated foams, are relatively stable and bio-compatible, but with intriguing molecular structures that reveal a new class of surfactant activity. Here we review the structures and functional mechanisms of some of these proteins as revealed by experiments involving a range of biophysical and biochemical techniques, with additional mechanistic support coming from more recent site-directed mutagenesis studies.

## Highlights

• Biological foams contain a cocktail of unusual proteins with diverse properties.
• Natural foam proteins have surfactant properties equal to or better than conventional detergents.
• They reveal new physical principles based on conformational change at interfaces.
• They illustrate alternative surfactant mechanisms not available to conventional detergents.
• Can act synergistically to form and stabilize bio-compatible, hydrated foam structures.

## Graphical abstract

**Figure:**
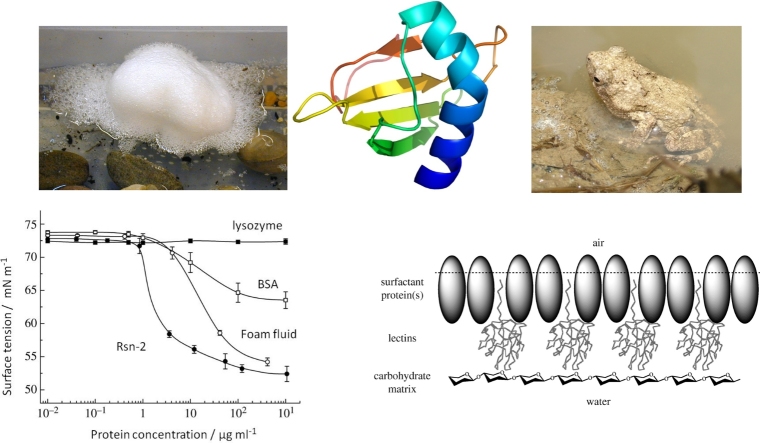

## 1 Introduction

The mechanical agitation and surfactant activities normally required to create aqueous foams and emulsions are generally damaging to biological systems. Strong shearing forces and interactions at surfaces and other interfaces can disrupt delicate cells and tissues, and can also denature proteins and other biological macromolecules. Conventional ionic/non-ionic detergents and surfactants will disrupt the phospholipid bilayers of cell membranes and can denature proteins. Indeed, various combinations of mechanical disruption and detergent extraction have long been used in laboratory procedures for the homogenization of biological tissues and the extraction and analysis of their various components for biochemical research and other purposes. Consequently, it is perhaps not surprising that natural foams and surfactants are relatively rare in biology. However, there are a few instances where natural biological foams and surfactants have evolved for apparently quite specific purposes, presenting us with an opportunity to explore potentially interesting new physics and physical chemistry of foams in an unusual context. Here we review some of our recent work in Glasgow, focussing mainly on the structure and function of some of the proteins that we have identified and characterized as a result of fieldwork and collaborations with colleagues worldwide [1], [2], [3], [4], [5], [6], [7]. Work on other surfactant proteins has been reviewed elsewhere [1], [8].

It is a pleasing historical coincidence that some of the early work on foam physics was initiated in Glasgow by William Thomson (Lord Kelvin). It was here that he devised the classic polyhedral structural model for foams as efficient space-filling entities [9] that has only more recently been superseded by the work of the Dublin group in the form of the Weaire-Phelan model [10], [11]. Our own interest in biological foams arises out of a natural curiosity for some of the natural foams, in particular frog foam nests, which had not been previously investigated.

**Fig. 1:**
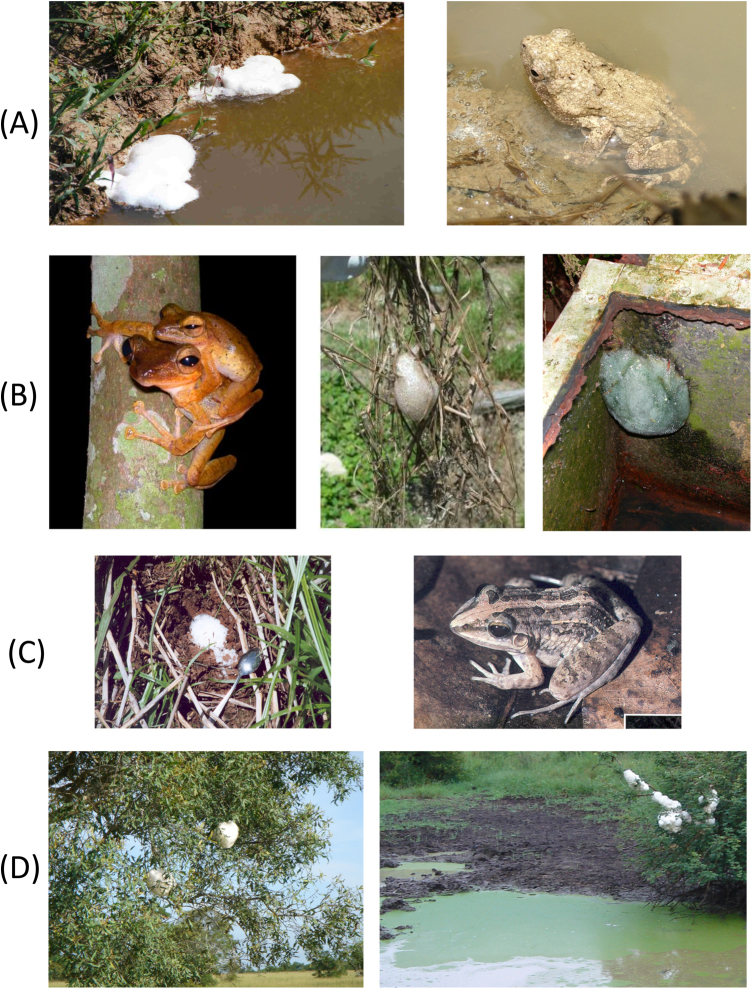
Frog foam nests from different continents. (A) Trinidad: *Engystomops pustulosus* (“túngara” or “mud puddle frog”). (B) Malaysia: *Polypedates leucomystax* (Java whipping frog). (C) Trinidad: *Leptodactylus fuscus* (whistling frog). (D) South Africa: *Chiromantis xerampelina* (grey foam-nest tree frog).

Foam nesting is one of the strategies that have evolved to allow some species of frogs to provide an appropriate environment for their eggs and embryos in regions of the world where standing water is otherwise rare or transient (Fig. 1). These nests are typically found in temporary pools or puddles (Fig. 1A), on tree branches or other structures overhanging water (Fig. 1B and D), or buried underground (Fig. 1C). As we have shown in earlier work [1], [2], [3], [4], these water-based foams are made from dilute solutions of proteins and carbohydrates that are very stable under tropical conditions. They resist dehydration, predation, and microbial degradation, and provide a biocompatible environment for the frog eggs, sperm and developing embryos. As an interesting form of soft matter they merit curiosity-driven investigation in their own right, but they also suggest numerous opportunities for potential practical application. For example, their biocompatibility and moisture retention, coupled with possible antimicrobial resistance suggests possible biomedical applications including use as temporary wound or burn dressings, surgical cavity filling or matrix for tissue regeneration, or slow-release drug delivery systems. In biotechnology, the surfactant proteins in these foams might be utilized for interfacing to nanoparticle/nanotube/graphene-based components and devices. On a larger scale, as biodegradable aqueous foams they might find application in the treatment of oil spillages, the bioremediation of contaminated areas, or other environmental issues. Plus, as we will indicate later, the surfactant activities of these foam components can help disperse carbon-based nanoparticles, with potential toxicology implications [12].

But, of course, in order to implement such potential applications it is necessary to explore more fully the chemical composition and molecular structures of the foam materials. Here we shall present some specific examples, describing how details about molecular structures and composition are leading to new insights into biological foam.

## 2 Ranaspumins

**Fig. 2:**
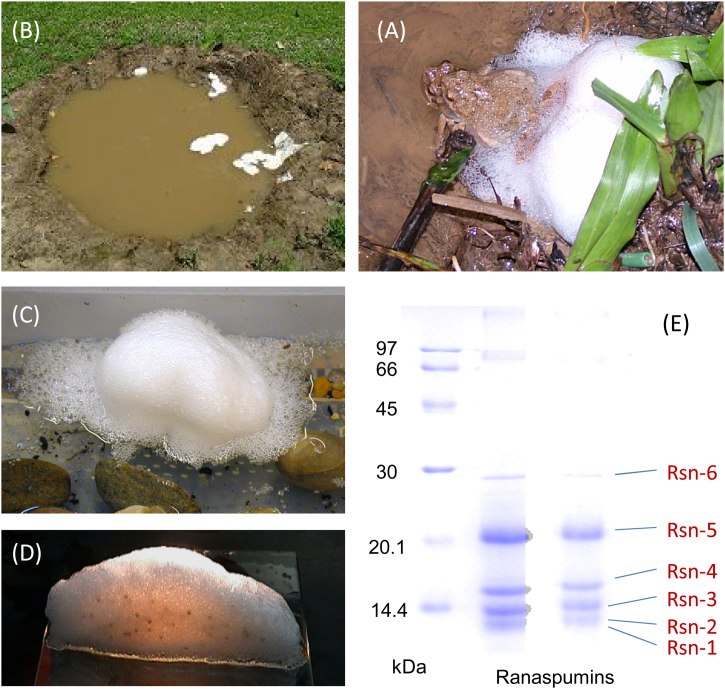
Foam nests of the túngara frog (*E. pustulosus*). (A) Nesting pair in amplexus. (B) Colony nests in a temporary pool (Lopinot valley, Trinidad). (C) Single nest, *ca.* 10 cm diameter, sliced through (D) showing the distribution of eggs (*ca.* 400) within the foam. (E) SDS gel electrophoresis analysis of foam fluid showing six major protein components (ranaspumins), standard molecular weight (kDa) markers to the left. (Picture credits: Alan Cooper, Rachel Fleming).

The túngara or mud-puddle frog (*Engystomops pustulosus*, previously known as *Physalaemus pustulosus*) is a common ground-dwelling foam-nesting species of Trinidad and surrounding areas of the Caribbean and Central America. Nests are formed during rainy seasons at the edges of temporary puddles or other standing water to provide a stable environment for the developing eggs and embryos (Fig. 1, Fig. 2).

During amplexus, the female repeatedly produces a small batches of eggs along with fluid that the male gathers up, fertilises and, with an egg-beater like motion of the hind legs, whips up to create the foam nest (see [13] supplementary data for videos of this process). Nest building occurs in discrete phases, the first of which is to produce a bubble raft, followed by the construction of a hemispherical foam mass into the centre of which the fertilised eggs are deposited [13]. Depending on conditions, tadpoles develop in the nest over the next few days before escaping into surrounding water, if available. The foam becomes dispersed when the tadpoles hatch, but if the eggs are removed at an early stage then the foam mass remains stable for many days under natural tropical conditions. The nests have typical wet-foam bubble morphologies, comprising 90% air, with the aqueous phase made up of a dilute solution of proteins (1–2 mg ml^−1^) together with a similar concentration of complex, long-chain carbohydrate molecules [2], [3].

**Fig. 3:**
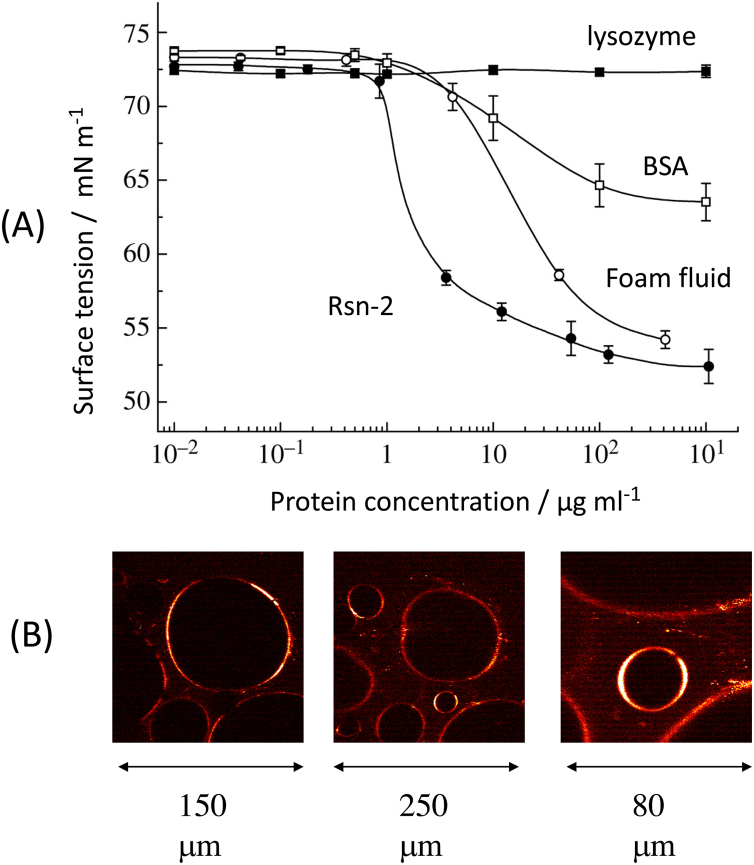
Surfactant properties of *E. pustulosus* foam. (A) Aqueous surface tension versus concentration of natural foam fluid compared to pure Rsn-2 and control proteins (BSA, lysozyme). (B) 2-photon fluorescence microscopy imaging of bubbles in frog nest foam.

Despite the fact that we have found no evidence for fats, lipids, or small detergent-like components in this foam fluid, the natural material has significant aqueous surfactant properties. Natural foam fluid can wet hydrophobic surfaces and reduces surface tension at (total) protein concentrations as low as 10–100 μg ml^−1^ (Fig. 3), much more effectively than proteins such as lysozyme or bovine serum albumin (BSA) that are not normally surfactant in their native states. Biochemical analysis shows that the fluid is made up of a cocktail of proteins, of which six predominate (the “ranaspumins”, Rsn-1 to Rsn-6) in the 11–25 kDa size range with intriguing properties. Some, at least, of these proteins can bind hydrophobic fluorescent molecules, and this can be used in 2-photon microscopy techniques to image bubbles in the foam [2] (Fig. 3). Amino acid and DNA sequence analysis showed little or no immediate similarity to previously known proteins, but with some potential clues as to their possible function.

### 2.1 Ranaspumin-2

One protein in particular, ranaspumin-2 (Rsn-2), has a particularly unusual and (so far) unique amino acid sequence, with a highly-charged hydrophilic C-terminus coupled with a significantly non-polar N-terminal sequence that is immediately suggestive of the amphiphilic characteristics of common detergents, but on a much larger molecular scale. Surfactant measurements using pure recombinant Rsn-2 show that this protein is indeed very effective at reducing the surface tension of water at very low concentrations (1–10 μg ml^−1^), an order-of-magnitude lower than the natural protein mixture (Fig. 3). And dilute solutions of Rsn-2 can be whipped up to produce foams that appear superficially similar to those seen in natural foam nests, albeit that they tend to collapse more rapidly (in a few hours) than the natural foams (days/weeks).

**Fig. 4:**
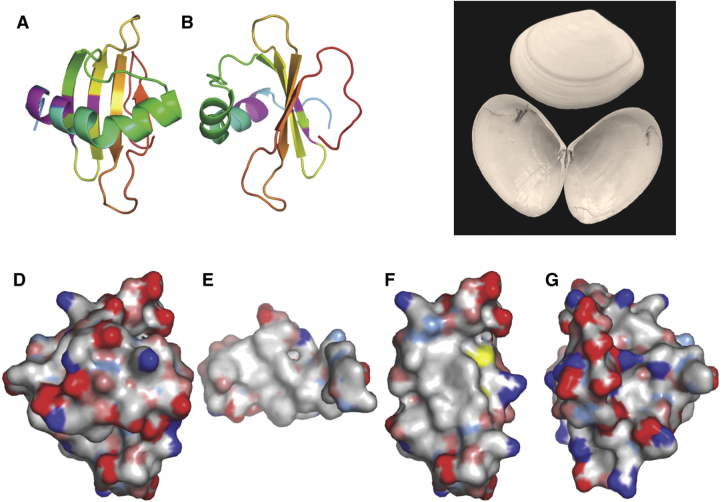
Solution structure of recombinant ranaspumin-2 as determined by NMR. (A) Ribbon diagram illustrating the Rsn-2 fold and (B) rotated 90° about the vertical axis, suggesting (C) a clam-shell opening mechanism. (D–G) Hydrophobic surface maps of the entire Rsn-2 molecule, front face (D) and back face (G), with the inner face of the helical segment (E) and the inner face of the sheet segment (F). Color codes: white, hydrophobic; red, negative; blue, positive; and yellow, sulphur. Adapted from [4]. (For interpretation of the references to colour in this figure legend, the reader is referred to the web version of this article.)

What is the three-dimensional structure of Rsn-2 that might explain these properties? Using high-resolution nuclear magnetic resonance (NMR) techniques on isotope-enriched samples of recombinant Rsn-2 [4], we have shown that its structure in water is monomeric and typical of a compact, well-folded and highly soluble globular protein (Fig. 4). Surprisingly, despite the amphiphilic nature of the extended polypeptide chain, the NMR structure of the Rsn-2 molecule in bulk aqueous solution shows no obvious hydrophobic patches or other structural features that might be anticipated for a macromolecular surfactant. (In this respect it differs significantly from other surfactant proteins such as hydrophobins [14] that tend to aggregate in solution.) This leads to the suggestion that Rsn-2 might undergo significant conformational change at the air-water or other non-polar interface. Closer inspection of the Rsn-2 structure shows that it is made up of two compact domains, linked by a potential hinge that might allow for a clam-shell-like opening of the structure to present hydrophobic faces of each domain to the interface, whilst maintaining much more polar regions in contact with the aqueous phase. This novel surfactant mechanism neatly solves the conundrum of how to achieve surfactant properties in a macromolecule whilst retaining a compact structure and high solubility in bulk water. It also addresses the issue of biocompatibility, since such large soluble protein molecules are less likely to penetrate and disrupt lipid bilayer membranes in the way that small molecule detergents do.

**Fig. 5:**
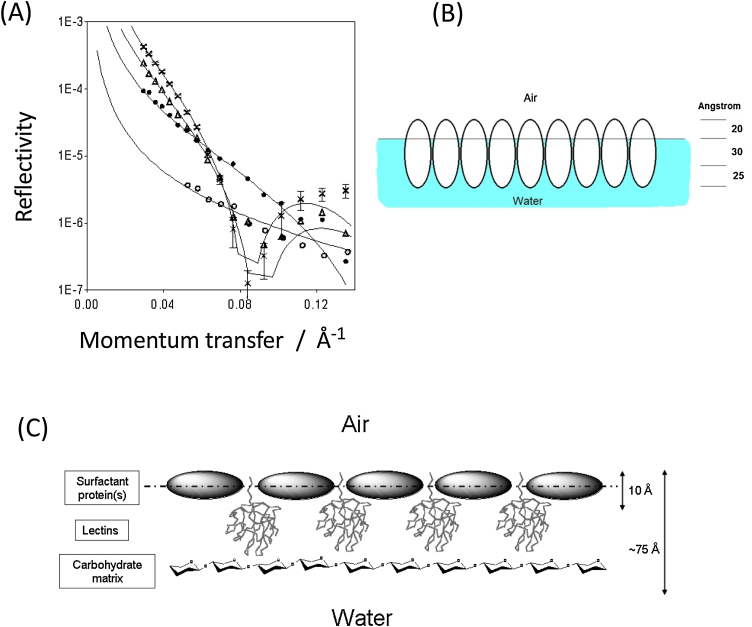
(A) Small angle neutron reflection profiles of the natural túngara frog foam mixture. (B) These are consistent with a 3-layer model that, together with additional information, suggests (C) a stable surface structure incorporating surfactant proteins, lectins and polysaccharides.

**Fig. 6:**
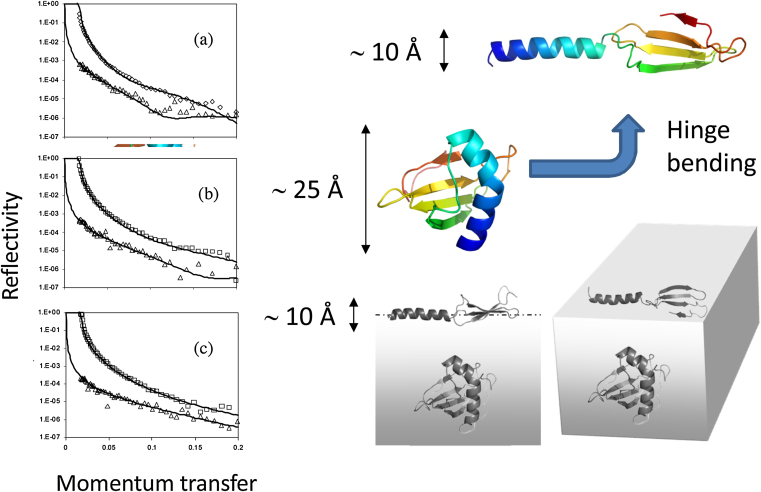
(Left) Neutron reflectivity data for Rsn-2 at the air-water interface in D_2_O (□, ◊) or null reflecting water (NRW) (Δ) at pH 7 and protein concentrations of (a) 0.25, (b) 0.05, and (c) 0.007 mg ml^−1^ respectively. These data are consistent with a narrow interface layer, compatible with the hinge bending model (right).

Although it is not (yet) possible to determine detailed molecular structures of proteins at interfaces, there are several lines of experimental evidence that lend support to the hinge-bending clam-shell model for Rsn-2 surfactant activity. Small-angle neutron reflection (SANR) experiments on the natural foam fluid [2] show a complex air-water interface layer, about 7.5 nm (75 Å) thick, probably made up of several regions of different protein/carbohydrate composition (Fig. 5). Similar experiments with pure Rsn-2 reveal a much simpler 1 nm (10 Å) monolayer (Fig. 6) [4]. This is narrower than would be expected for Rsn-2 in its compact, folded form (diameter *ca*. 2.5 nm), but is compatible with the proposed open clam shell structure at the interface. Infra-red reflection absorption spectroscopy (IRRAS) of characteristic protein amide-I and amide-II bands shows that Rsn-2 retains the anticipated α-helix and β-sheet secondary structure at the air-water interface, with an angular dependence consistent with a planar orientation in the interfacial layer [4].

**Fig. 7:**
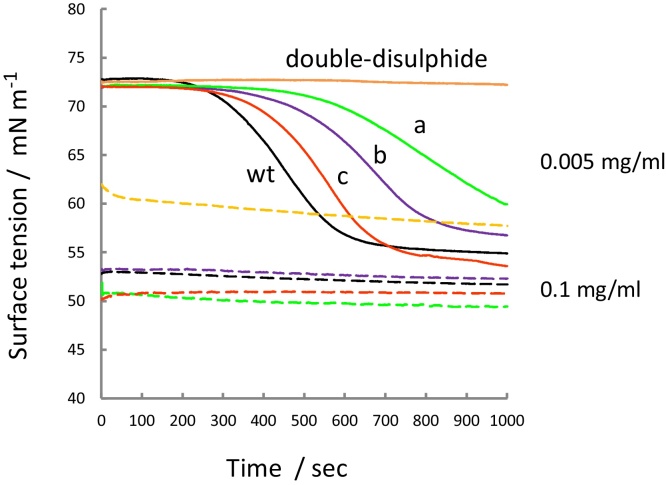
Development of surface tension over time for the Rsn-2 disulphide mutants at two different concentrations: 0.005 mg ml^−1^ (solid lines) and 0.1 mg ml^−1^ (dashed lines), compared to the wild type (wt) protein at the same concentrations. Note that surfactant activity is almost totally suppressed in the double-disulphide mutant (N19C-D32C-I46C-M81C), and is reduced in the case of the other mutants D32C-M81C (a), N19C-I46C (b), and C68T-C86A (c). See [15] for details.

In more recent work we have used site-directed mutagenesis to introduce chemical modifications into the Rsn-2 structure in order to investigate the surfactant mechanism [15]. Chemical cross-links can be inserted into the structure by pair-wise conversion of selected amino acids to cysteine residues, thereby forming disulphide bonds that might block, or at least partially compromise the hinge opening mechanism. Results from a series of Rsn-2 disulphide mutants are illustrated in Fig. 7, showing that in all cases the onset of surfactant activity, as measured by surface tension, is eliminated or significantly reduced in ways that are consistent with inhibition of hinge opening. In other experiments [16], modification or removal of portions of the hydrophobic N-terminal sequence resulted in diminution or loss of surfactant activity, suggesting that insertion of this end of the polypeptide chain into the non-polar interface provides some of the driving force for the conformational change mechanism. C-terminal modifications show lesser effects, though reductions in the rates of onset of surfactant activity were observed [15], [16].

Indirect evidence for the hinge-bending mechanism comes from molecular dynamics simulations of what might happen as an Rsn-2 molecule in water approaches a non-polar interface [16], [17]. Consistent with experimental observations, these simulations show that Rsn-2 may adsorb at an air-water interface, with the majority of the non-polar N-terminal residues exposed to the air. The protein secondary structure (α-helix, β-sheet) remains mostly unchanged in these simulations.

### 2.2 Other ranaspumins

Much less is known in detail about the other major proteins in the túngara frog foam nest. Comparison of amino acid sequences suggests that Rsn-1 (like Rsn-2) might be related to cystatins [15], a class of protease inhibitors that can block the breakdown of protein polypeptides by enzymic degradation. Indeed, in preliminary experiments we have demonstrated potent protease inhibition activity in natural foam material, though this has not yet been pinned down to any specific component in the foam. Recombinant Rsn-1 also reduces aqueous surface tension, though not quite as effectively as Rsn-2 [15]. Other ranaspumins in the mix (Rsn-3 to Rsn-6) can be identified as carbohydrate binding proteins (lectins) [3], and this may be related to the possible role of the complex, long-chain carbohydrates in the foam cocktail. Lectin activity and carbohydrate binding has been confirmed in one case (Rsn-4) by demonstration of red blood cell agglutination that is inhibited by lactose and galactose [3].

From work done so far it would appear that foam nest proteins from other frog species have little in common. Preliminary analysis of proteins from the foam nests of other species, including *Leptodactylus fuscus* (Central and South America), *Limnodynastes peronii* (Australasia) and the Asiatic tree-nesting species *Polypedates leucomystax* and *Rhacophorus arboreus*, shows no resemblance to the túngara ranaspumins [3]. Likewise, recent structural studies by others [18] of a putative surfactant protein (Lv-RSN-1) from another South American species, *Leptodactylus vastus*, again shows no similarity to the túngara proteins at either the amino acid sequence or protein conformational level, although it does share some sequence similarity with a nest protein from *L. fuscus* [3], [18] and also appears to have a very hydrophobic, unstructured N-terminus that might be related to surfactant function. This general diversity in surfactant protein structure and function has also been noted elsewhere [8], suggesting that these proteins do not necessarily share a common evolutionary heritage.

### 2.3 Foam nest stability

As mentioned above, dilute solutions of pure recombinant Rsn-2 can be whipped up to create foam superficially identical to that seen in natural foam nests. However, these foams in the lab have relatively short term stability, usually collapsing within an hour or so. This is in marked contrast to what we observe in the wild, where natural frog foam nests survive for many days, and where subsequent collapse or degradation is normally due to mechanical disruption or enzymatic degradation by the tadpoles themselves rather than underlying physics. This suggests that there must be additional factors present in the natural nests that contribute to longer term stability.

These ranaspumin-based aqueous foam liquids are very dilute and low viscosity, so stability is unlikely to arise from viscous bubble-entrapment/kinetic effects, at least not in this particular species. The stabilization of foams and emulsions by incorporation of small particles has been described in numerous other (mostly non-biological) systems [19], [20], but again this is unlikely to be a major factor here. Inevitably, given the nature of the biological environment, natural foam nests often contain particulate debris picked up from the surroundings. But this is quite adventitious and varies from site-to-site. Microscopic examination of foam fluid does reveal the presence of small particles (and micro-organisms), but not at a level likely to be relevant to foam stabilization. Moreover, our laboratory-based experiments with captive frogs in pure, de-ionized water show that equally stable foam nests can be produced in the absence of extrinsic factors.

The answer to the stability puzzle must lie in the intrinsic composition of the foam nest cocktail of proteins and carbohydrates. Our current working hypothesis, illustrated in Fig. 4, is that the interfacial layer in the natural foams incorporates other proteins and carbohydrates to form a more stable system. Initial bubble formation is facilitated by the surfactant activity of Rsn-2, but subsequent addition of lectins (Rsn-3/4/5/6?) builds up a more complex layer that may then also be reinforced by binding to long-chain branched polysaccharide molecules. At least two of the ranaspumin lectins (Rsn-3 and Rsn-5) have amino acid sequences possessing moderately hydrophobic [3] N-terminal tails that might serve to anchor and orient these molecules in the interface. Further binding to an underlying carbohydrate layer would create a mechanically stable, water-retaining foam matrix with a thickness compatible with the SANR data.

It should be borne in mind that foam nest stability in the wild will depend on factors other than plain physics − in particular, resistance to predation and microbial degradation is important in the biological context. Lectins similar to Rsn-3/4/5/6 can inhibit microbial growth by agglutination of cells and can also act as anti-feedants in higher organisms [3]. Lectins and protease inhibitors in plants [21] and detergent secretions [22] can act as defences against insect attack. It seems likely, therefore, that a combination of these properties found in the nest proteins are sufficient, acting synergistically, to stabilise the nest against environmental and biological challenges for just long enough for the purpose of tadpole development without replenishment.

## 3 Ranasmurfin

**Fig. 8:**
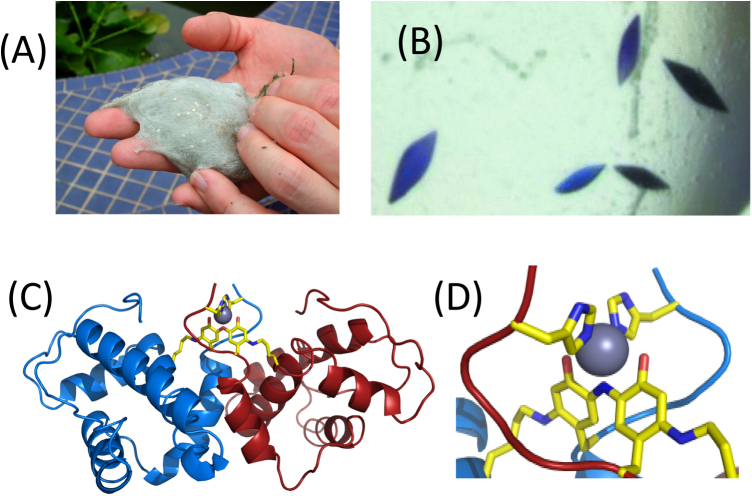
Ranasmurfin: protein extracted from pigmented foam nest (A) forms intense blue crystals (B) suitable for X-ray crystallographic structure determination [6]. The protein is a dimer (C) stabilized by numerous post-translational cross-link modifications, including an unusual and unprecedented Zn-chelating four-residue (Lys-Tyr-N-Tyr-Lys) linkage between the two Rsf subunits (D) that is also responsible for the blue colour.

Many tree frogs also build foam nests, usually overhanging water on branches or other adjacent structures. One example of this is *P. leucomystax*, a common tree frog from Malaysia and surrounding regions, whose nests have the intriguing property of developing a blue/green pigmentation over time (Fig. 1, Fig. 8) [6]. This coloration is due to an unusual zinc-containing protein, designated ranasmurfin (Rsf), which crystallises readily after purification from natural nest material. X-ray diffraction from these intense blue crystals was used to determine the protein structure to high resolution, revealing a 26 kDa dimeric structure incorporating various unusual chemical modifications (Fig. 8) [6].

The function of this protein is not yet known, but it is present in the foam at relatively high levels suggesting that it may be involved, at least in part, in foam stabilization, adhesion, or other mechanical properties, possibly utilising its chemical cross-linking properties. Other possibilities include camouflage and/or UV protection. The total protein concentration in *P. leucomystax* foam fluid is 2–4 mg ml^−1^, depending on sample, with about 1–1.5 mg ml^−1^ carbohydrate (Rosalind Tan, Malcolm Kennedy & Alan Cooper, unpublished). The foam itself is rather sticky and viscous in this species, and is probably kinetically stabilised by viscous incorporation of bubbles rather than by specific surfactants.

## 4 Latherin

**Fig. 9:**
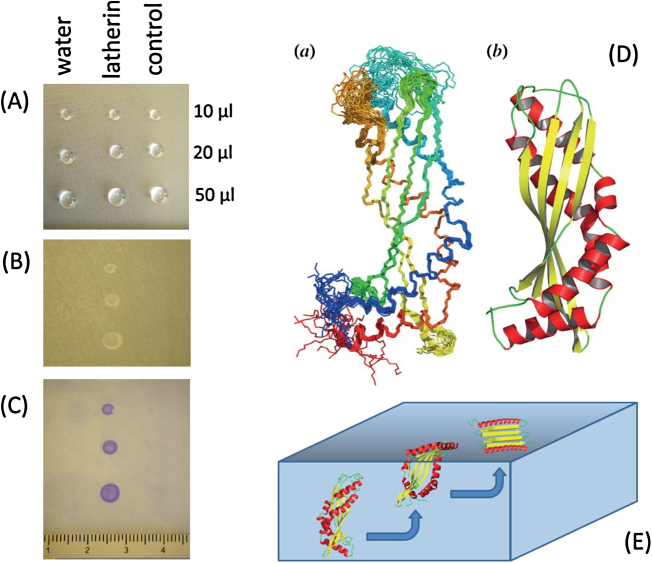
Latherin wetting and adsorption to hydrophobic surface, in comparison with water and protein (lysozyme) controls: (A) 10, 20 and 50 μl drops, 1 mg/ml, on Nescofilm sheet, followed by rinsing (B), drying and (C) staining for adsorbed protein [5]. (D) The solution structure of latherin: (a) ensemble of 20 latherin models (superimposed) that best fit the experimental NMR data, shown in peptide backbone representation; (b) ribbon model of the representative structure illustrating secondary structure elements. (E) Unfolding model for surface activity. Adapted from [7].

Latherin is a protein found in the sweat of horses and is one of the first proteins shown to have strong surfactant activity in its native state [23] and adsorbs well to waxy surfaces (Fig. 9). Its function is thought to be to act as a wetting agent to facilitate evaporative cooling from the oily surface of the pelt [5], [23] where it is probably the contributing factor to the foaming/frothing of horse sweat during vigorous exercise. It is also found in horse saliva and is a member of the palate, lung, nasal epithelium clone (PLUNC) family of innate immunity proteins that are abundant in the oral cavity and saliva of mammals [5], [7] and which have surfactant, bacteriostatic and antibiofilm properties [24]. Latherin is characterized by an unusually high content of non-polar amino acids, especially leucine which makes up about 24% of the latherin total, compared to an average of around 10% for most other proteins.

Although such abundance of non-polar amino acids might be thought to be the source of latherin surfactant activity, this is not immediately apparent in its three-dimensional structure in solution (Fig. 9). The protein is a monomer in solution, with a structure, as determined by NMR [7], comprising a slightly curved cylindrical structure in a ‘super-roll’ motif made up of a four-stranded anti-parallel β-sheet and two opposing α-helices which twist along the long axis of the cylinder. Although there is no obvious hydrophobic patch on the protein surface, one end of the molecule does have prominent, flexible polypeptide loops that contain a number of apolar amino acid side chains. This suggests a plausible mechanism for surfactant activity in which the molecule is first localized to the non-polar interface via these loops, and then unfolds and flattens to expose its hydrophobic interior to the air or non-polar surface (Fig. 9). As was also the case with Rsn-2, this is supported by SANR experiments that indicate that latherin forms a thin, ca. 1 nm (10 Å) air-water interface layer consistent with unwrapping or unfolding of the protein [5], [7].

## 5 Applications and implications

**Fig. 10:**
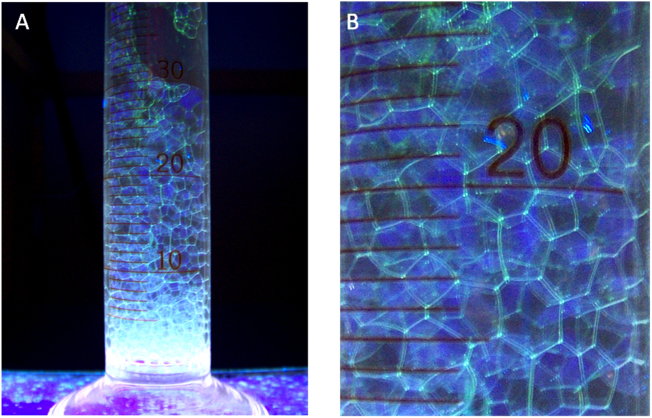
Fluorescence under UV illumination of the Rsn-2/iLov construct. (A) Foam was generated in a 50 ml glass measuring cylinder by bubbling air through a solution of Rsn-2/iLov at a concentration of 1 mg ml^−1^ in aqueous phosphate buffer, pH 7.5. (B) Close-up of the individual bubbles [15].

Despite the promising range of potential applications mentioned in the Introduction, it is still early days, and relatively few examples exploiting natural protein surfactants have been reported so far. Applications in commercial biomedical, food technology and related areas may be hindered by a reluctance to embark on the necessary investigation of potential toxicology and allergenicity implications for these biomaterials from unusual sources. Nonetheless, other potential applications are making progress. Recombinant Rsn-2 has been used by others to form a stable foam matrix incorporating components for a cell-free artificial photosynthesis system [25]. In unrelated work, we have taken advantage of recombinant protein technology to construct a fluorescent Rsn-2/iLov protein conjugate (Fig. 10) [15], demonstrating how the surfactant properties can be attached to other functional proteins.

**Fig. 11:**
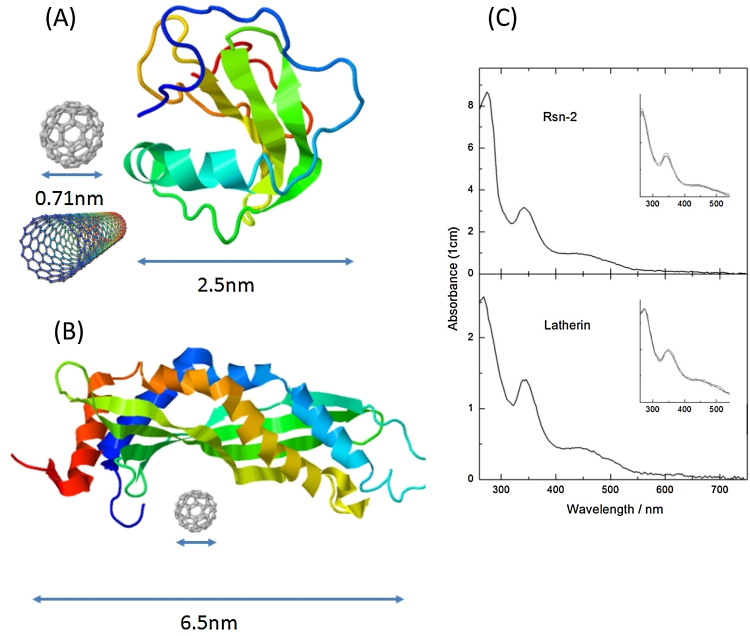
Left: cartoons illustrating the size of fullerene C_60_ and carbon nanotube in comparison to the surfactant proteins Rsn-2 (A) and latherin (B), approximately to scale. The structures for Rsn-2 (pdb: 2WGO) and latherin (pdb: 3ZPM) are taken from [4] and [7], respectively. Right: (C) UV–vis spectra of C_60_ dispersed in aqueous solutions of Rsn-2 (upper panel) or latherin (lower panel). The inserts show superimposed spectra of samples after storage at 4 °C for approximately 1, 2 and 6 months [12].

This ability to engineer recombinant proteins is particularly relevant for possible applications in nanotechnology involving interface of biological macromolecules to other components. For example, as we have recently shown [12], both latherin and Rsn-2 can be used to solubilize or disperse C_60_-fullerene and bind to carbon nanotubes in water (Fig. 11). The next step (work in progress) is to utilize this binding to attach other functional proteins to the carbon (nanotube, graphene) surface.

The aqueous dispersion/solubilization of hydrophobic particles by natural surfactant proteins demonstrated here has potential toxicology implications, indicating a possible route for biological uptake of carbon nanotubes and other nanoparticles [12]. This might, for instance, be of potential concern for nanoparticle uptake by proteins related to latherin (PLUNCs) that are present in the human respiratory tract and elsewhere.

## 6 Experimental

Methods for the preparation and characterization of natural surfactant proteins have been described in detail in previous reports [2], [3], [4], [5], [6], [7], [15], so will be briefly summarized here. Ranaspumins were initially identified by gel electrophoresis of foam nest material of the túngara frog (*E.* pustulosus) collected in Trinidad. Partial amino acid sequencing of these proteins facilitated isolation of mRNA from frog oviduct tissue and subsequent complementary DNA sequencing. Recombinant DNA methods were then used to construct a bacterial (*E. coli*) clone producing Rsn-2. Isotope-labelled samples for NMR structure analysis were prepared by growing these bacteria in ^13^C/^15^N-enriched media. Site-directed mutagenesis techniques were used to prepare sequence-modified and truncated proteins [15], [16]. More specifically, for the Rsn-2 disulfide cross-linked mutants newly described here, appropriate pairs of adjacent amino acid residues were identified from the protein structure and converted into cysteine residues using standard site-directed mutagenesis [15]. Two single disulphide (N19C-I46C and D32C-M81C) and one double-disulphide (N19C-D32C-I46C-M81C) Rsn-2 mutants were produced in this way for comparison with the wild-type (wt) protein. One further mutant (C68T-C86A) was constructed in order to remove the existing native disulphide for comparison. Circular dichroism spectroscopy (CD) was used to show that the secondary structure composition had not been significantly affected by the introduced mutations [15]. Latherin was produced by similar recombinant protein expression and purification [7], [15].

Ranasmurfin was isolated by chromatography from foam nest material of the common Asian tree frog (*P. leucomystax*) collected in Malaysia, and its amino acid sequence was determined by a combination of high-resolution X-ray crystallography and *de novo* sequencing using mass spectrometry [6].

Protein structures were determined by solution NMR (Rsn-2, latherin) or X-ray crystallography (ranaspumin). Air-water interface properties were measured using small-angle neutron reflection (SANR) [2], [4], [5] and infra red reflection absorption spectroscopy (IRRAS) [4] methods, together with standard Langmuir trough and surface tensiometer techniques.

## 7 Summary

• The components of frog foam and horse sweat have natural surfactant properties equal to or better than conventional detergents and other surfactant proteins on a molar basis.
• These materials display a wealth of new proteins with additional properties such as protease inhibition and carbohydrate binding.
• They illustrate alternative surfactant mechanisms not available to conventional detergents, involving conformational change at interfaces.
• Fascinating physics, the understanding of which must go hand in hand with the biology.
• These new proteins – together or separately – contribute to the formation and stabilization of bio-compatible, hydrated foam structures.
• Multiple potential applications, yet to be exploited.
